# Necrotizing Lymphocytic Vasculitis Limited to the Peripheral Nerves: Report of Six Cases and Review

**DOI:** 10.1155/2009/368032

**Published:** 2010-03-01

**Authors:** José Félix Restrepo, Federico Rondón, Eric L. Matteson, Carlos H. Colegial, Gerardo Quintana, Antonio Iglesias-Gamarra

**Affiliations:** ^1^Facultad de Medicina, Universidad Nacional de Colombia, Calle 45, Cra 30, Bogota, Colombia; ^2^Division of Rheumatology, Mayo Clinic College of Medicine, 200 1st Street SW, Rochester, MN 55905, USA

## Abstract

*Background*. The systemic vasculitides are syndromes characterized by inflammation and injury (necrosis or thrombosis) of blood vessels, resulting in clinical manifestations according to the affected vascular bed, but not classically in stocking-glove neuropathy. *Objective*. To describe a form of primary vasculitis affecting strictly peripheral nerves manifesting as stocking-glove neuropathy. *Methods*. Case series of 110 patients seen in three centers in Bogotá who presented with symptoms and signs of polyneuropathy and/or were identified with vasculitis affecting only the peripheral nerves, and who underwent sural nerve biopsy. *Results*. Six patients had a vasculitis affecting only the peripheral nerves diagnosed on sural nerve biopsy which demonstrated a mixed infiltrate of monocytes/macrophages and lymphocytes especially in the small epineurial blood vessels. Over time, all had worsening of symptoms, with grip weakness and motor deficits in the hand and feet. Serologies and acute phase reactants were normal in all patients. Treatment response to immunosuppression was satisfactory in 5 patients; 1 patient had progressive neurologic damage. *Conclusions*. There is a distinct form of primary vasculitis of the peripheral nervous system characterized by distal sensory polyneuropathy with stocking-glove distribution with good prognosis, few and minor relapses and good response to treatment even after delayed diagnosis.

## 1. Introduction

The systemic vasculitides are clinically heterogeneous entities characterized by inflammation and damage by necrosis or thrombosis of blood vessels, with clinical manifestations according to the distribution of the involved vessels [1]. 

 Peripheral neuropathies have associated with or been attributed to the presence of vasculitis in a variable proportion of patients, including infections, myeloproliferative disorders, and many inflammatory conditions. In some series, up to 75% of peripheral neuropathies occurring in patients with connective tissue diseases and systemic vasculitis are due to vasculitis [2, 3]. The peripheral neuropathy of most of these disorders is usually due to mononeuritis multiplex or sensorimotor polyneuropathy and can be difficult to clinically distinguish from other causes.

The purpose of this study is to describe the presence of a primary vasculitis affecting only the peripheral nerve and clinically manifesting as a stocking-glove neuropathy, its clinical course, associated clinical findings, and discuss its pathogenesis.

## 2. Materials and Methods

We performed a study of all 110 patients presenting between 1993–2004 with symptoms and signs of polyneuropathy or who presented at electromyography (EMG) with mononeuritis multiplex or distal sensory-motor polyneuropathy suggestive of a primary or secondary vasculitis or vasculitis strictly confined to the peripheral nerves that underwent sural nerve biopsy. Demyelinating neuropathies were excluded. 

We employed the following definitions in establishing a diagnosis of necrotizing angiopathy of the peripheral nervous system:

1. segmental vessel wall necrosis of epineurial vessels,
2. inflammatory cell infiltrates at the level of the transneurium of the epineurial vessels,
3. fibrin deposition with or without thrombosis at the level of the vessels of the epineurium, and in some cases deposits of hemosiderin easily visible,
4. recanalization.

We considered the diagnosis if all of the first three criteria were present; criterion 4 may or may not have been present. Patients were classified as having lymphomonocytic vasculitis if there was presence of infiltrate composed mainly of mononuclear cells (monocytes, macrophages and lymphocytes), especially in arterioles and small sized vessels [4]. All patients underwent EMG according to a standard protocol [5]. 

Neuropathies were classified as mononeuritis multiplex (motor and/or sensory deficits in the distribution of one or more isolated peripheral nerve), distal symmetrical sensorimotor polyneuropathy (displaying symmetrical distal stocking-glove distribution of sensory and motor deficits), or asymmetrical/overlapping neuropathy (motor or sensory deficits with substantial limb asymmetry) [6].

All patients had a complete blood count (CBC), Westergren sedimentation rate, C-reactive protein, rheumatoid factor (RF), antinuclear antibody (ANA), anti-DNA, anti-RNP, anti-Ro, anti-La, anti-Smith, c-ANCA, p-ANCA, creatine phosphokinase (CPK), hepatitis B and C serologies, protein electrophoresis, C3 and C4 complement, glucose, total cholesterol and lipid profile, triglycerides, creatinine, AST, ALT, and chest radiograph.

## 3. Results

Of 110 patients who underwent sural nerve biopsy between 1993 and 2004, a total of 6 were diagnosed with vasculitis strictly confined to the peripheral nerves according to the criteria above. Five patients had sensorimotor axonal/demyelinating polyneuropathy and one patient had mononeuritis multiplex. Onset of symptoms was between 56 and 73 years of age (mean 64.8 years). All six patients were female. None fulfilled American College of Rheumatology or relevant other criteria for connective tissue diseases including systemic lupus erythematosus (SLE) [7], primary Sjögren syndrome [8], scleroderma [9], polyarteritis nodosa (PAN) [10], microscopic polyangiitis, Wegener's granulomatosis or Churg-Strauss disease [11]. The other diagnosis included 20 patients with RA, 25 SLE, 18 Sjögren syndrome, 8 polymyositis, 5 PAN, 3 mixed connective tissue disease, 12 undifferentiated connective tissue disease, 2 scleroderma, 1 case of lepra and the remainder had no other identifiable underlying disorder including cryoglobulinemia, malignancy, hepatitis B or C, toxin exposure, diabetes or other metabolic or nutritional disorders. 

### 3.1. Clinical Aspects

In all 6 cases the clinical picture developed slowly, with patients developing symptoms between 18 and 38 months before the diagnosis was made. At disease outset, patients sought attention for paresthesias, dysesthesias or pain in the hands and feet and symmetric lower extremity muscle weakness. Symptoms were commonly thought to represent entrapment neuropathies of the upper or lower extremities or to represent the start of the muscle inflammation of dermatomyositis/polymyositis (cases 5 and 6). After 3 to 6 months, signs of neurological compromise accelerated with worsening pain.

In a second phase of disease, patients presented with grip strength weakness and motor deficits in the hands and feet raising the possibility of a polyneuropathy. This was the point at which EMG was obtained. 

The average number of months between the onset of symptoms until EMG diagnosis in the 5 patients with sensorimotor axonal/demyelinating polyneuropathy was 22.4 months and 18 months in the one case of mononeuritis multiplex. EMG involvement in order of frequency from high to low was external popliteal sciatic nerve, internal popliteal-sciatic nerve, sural nerve, median nerve and ulnar nerve. In the case of mononeuritis multiplex foot drop of the left foot was present.

**Figure 1:**
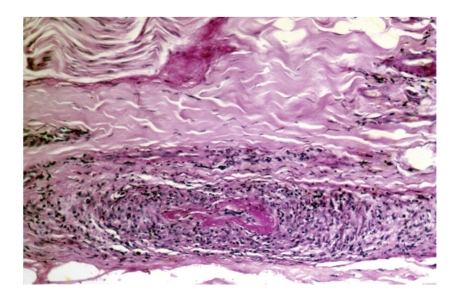
(Case  4). Medium diameter epineural artery. A prominent lymphocytic inflammatory infiltrate is present in the thickened vessel wall, with fibrinoid material causing luminal obstruction. H&E, 40X.

**Figure 2:**
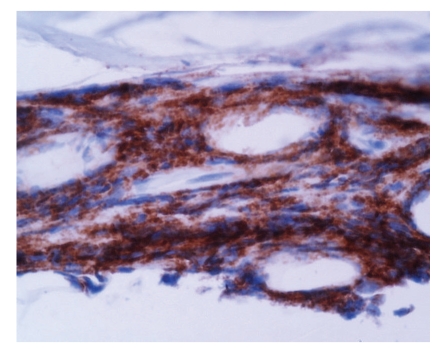
(Case  5). Immunoperoxidase staining with CD20, demonstrating the great majority of cells to be B lymphocytes, suggesting a humoral pathophysiologic diathesis. 40X.

The time lapse between the EMG study and the sural nerve biopsy in the 6 patients was between 45 and 60 days. The result of the sural nerve biopsy in all 6 patients was compatible with a monocytic/macrophage and lymphocytic infiltrate especially at the level of the arterioles and small caliber blood vessels. Findings were of a necrotizing lymphomonocytic vasculitis strictly confined to the epineurium of the peripheral nerves. The average period of observation after diagnosis was 4 years (range: 2.7 to 8 years), during which no manifestations suggestive of systemic involvement or other disease occurred (Figures 1 and 2). 

### 3.2. Laboratory Findings

The Westergren sedimentation rate (ESR) was modestly elevated in all cases at diagnosis (between 30 and 40 mm/hr; mean 34). ANA was detectable in two patients in low titer (≤1: 160), and anti-Ro, anti-La, anti-Smith, ant-RNP, RF, p- and c-ANCA, protein electrophoresis, C3 and C4 complement and markers of hepatitis B and C were negative. 

The 6 patients have a similar clinical picture with involvement of the distal nerves consistent with the clinical and pathological picture of an idiopathic vasculitis strictly affecting the peripheral nerves of delayed onset (Table 1). A predominate lymphocytic infiltrate was present in the small and medium sized epineurial arteries (Figure 1). In case 5, immunoperoxidase staining with CD20 demonstrated a majority of cells to be B lymphocytes (Figure 2).

### 3.3. Treatment

All patients received treatment with glucocorticosteroids at a dose of  .5 to 1 mg/kilogram/day until control of symptoms of pain and dysesthesia was achieved. The daily corticosteroid dose was tapered by 5 mg each day to 30 mg a day, and then by 2.5 mg each week to a maintenance dose of 2.5 mg daily for one year, and then discontinued. All patients received concomitant azathioprine at a dose of 100 mg a day for two years unless on cyclophosphamide. Patients 5 and 6 required bolus cyclophosphamide (500 mg/month) over 1 year due to the severity and course of the disease. Patients 5 and 6 were treated with cyclophosphamide beginning 3–6 months after initiation of therapy because of poor initial treatment response. 

Complete recovery was achieved in 5 patients by 6 months following initiation of treatment; only patient 5 had a severe course with neurological sequelae. All patients had quiescent disease during the subsequent 2 years of follow-up.

## 4. Discussion

We identified a total of 6 patients with sensorineural polyneuropathy, with axonal and myelin degeneration occurring in isolation, without associated connective tissue, metabolic, toxic, degenerative, infiltrative, or infectious disease. 

The neuropathy in these cases is distal in nature, with stocking-glove dysesthesias. Histopathology reveals foci of lymphomonocytic infiltrate. We believe that this necrotizing angiopathy occurs in isolation, without systemic features, at the level of the peripheral nerves.

In 1880, Leyden [12] reported on polyneuropathies of inflammatory character occurring in the absence of a connective tissue disease, infection, toxins, cancers and other identifiable associations. One of these was polyarteritis nodosa, described as a unique clinical entity in 1866 by Kussmaul and Maier [13].

In 1972, a group of investigators from the Mayo Clinic introduced classification of angiopathic neuropathy, and included sural nerve biopsy results in their considerations, especially in patients with rheumatoid arthritis (RA), polyarteritis nodosa (PAN), and then later other connective tissue diseases [14]. This opened the door for an improved pathophysiologic understanding of these conditions. Three years later, Dyck et al. [15] reported on 53 Mayo Clinic patients with a diagnosis of chronic inflammatory polyneuropathy developing over an average of 7.5 years. They described the histopathology of the sural nerve in these cases, noting a mononuclear cell infiltrated suggestive of an inflammatory process, and concluded that these patients did not have vasculitis, but rather a nonvasculitic chronic inflammatory condition.

Other reports of isolated necrotizing angiopathy have included those of Kissel et al. [6] (7 patients), and again Dyck et al. [16] who described a further 20 patients with peripheral nerve involvement, including 5 with definite vasculitis and 15 with a vasculopathy. The average length of evolution of symptoms was 11.5 years. This publication served to sharpen the concept of nonsystemic vasculitic neuropathy. Dyck concluded that peripheral neuropathy can occur secondary to a necrotizing angiopathy, and in some cases can be associated with a connective tissue disease, but can also be due to an isolated angiitis of the peripheral nervous system.

Peripheral nerve disease may be asymmetric and involve the muscle, sometimes with an aggressive and chronically relapsing clinical course [17–21]. Biopsies may demonstrate perivascular infiltrate; in one report, 76% of cases had involvement of the peripheral nerves and muscles, supporting the concept of isolated vasculitis of the peripheral nervous system [22]. 

Immunoglobulin deposits and C3 may be detected in a substantial number of specimens [21]. Acute phase reactants are elevated in 20%–65% of reported cases [18–21]. In our cohort the ESR was only moderately elevated, with a median of 34 mm/1 hr.

Sural nerve biopsy has become an important diagnostic procedure for evaluation of vasculitis since its introduction in 1975. Like others, we have made increasing use of this technique. Of the 6 cases reported here, 1 was of the 21 patients studied in 1992 who had isolated peripheral nerve involvement [1]. This patient served as our index case and led us to our observations in the subsequent 12 years. 

All of our patients received treatment with glucocorticosteroids ad moderate doses and azathioprine with good clinical response, based on the complete control of symptoms and the normalization of the EMG findings, except for the 5th and 6th patients who received bolus cyclophosphamide because of rapid clinical deterioration. Patient 6 remains without symptoms or sequelae 2 year following this treatment. The relatively rapid response to treatment despite the delayed diagnosis suggests that there is persistent low grade inflammation possibly at the level of the epineurium, with the import that isolated peripheral nervous system vasculitis is a clinically more indolent and benign disorder than PAN and other forms of vasculitis. 

 Of particular note is the presence of CD20 positive (B-cell) lymphocytes in case 5. These cells are generally not described as a prominent feature of systemic or localized vasculitis, but suggest a possible role for them in the pathogenesis of vasculitis which requires further investigation. This patient has not developed a malignancy, including B-cell lymphoma, during the follow-up period. 

The histological pattern in the 6 patients is characterized by a lymphomonocytic infiltrate affecting the small arterioles of the vasonervorum of the distal peripheral nerves of the hands and feet and absence of polymorphonuclear cells, which can be mild, moderate or severe and significant endothelial thickening. Also 4 out of the sample has necrotizing vasculitis. The clinical course of these 6 patients is similar, with no systemic disease and virtually no laboratory abnormalities. It is possible that lymphocytic infiltration seen on biopsy is a late feature of the arteritis, disease, but this is difficult to assess because in virtually all cases, biopsies are performed after the disease has been established for some time. 

Finally, we believe that there is a primary angiitis of the peripheral nervous system, that may present in one of two forms. One is the well defined arteritis and distal asymmetric polyneuropathy which can cause muscle involvement with an aggressive course with relapses in 38% of cases, increased acute phase reactants and which can occasionally be fatal [15]. The other is a distal sensory polyneuropathy with stocking-glove distribution with good prognosis, few and minor relapses, and good response to treatment, like the cases we have presented in this article.

No randomized clinical controlled trial of therapy has been done in patients with nonsystemic vasculitis neuropathy [23], but the majorities of retrospective data support the use of glucocorticoid and immunosuppressive. In refractory cases, it has been used intravenous immunoglobulin, mycophenolate, rituximab, infliximab, or alemtuzumab [24].

We suggest that patients with a polyneuropathy without apparent underlying disease should be suspected of having an isolated vasculitis of the peripheral nervous system. These patients should undergo diagnostic evaluation including EMG and measurement of acute phase reactants, and in appropriate cases (sural) nerve biopsy. Even after a protracted clinical course, the symptoms may respond to treatment with glucocorticosteroids and/or other immunosuppressive therapies. 

Limitations from our study are the small sample size. In addition, because this is a case series report, the improvement of patient was based in the clinical observation and criteria by the treating physician, however, improvement was documented objectively by EMG in all patients. Larger samples and case controlled studies are needed to confirm our findings.

## Figures and Tables

**Table 1:** Idiopathic vasculitis strictly affecting the peripheral nerves.

Patient	Age (years)	Clinical aspects	Time between symptom onset and diagnosis (months)	Sural nerve biopsy findings	Comments	Duration of follow-up after diagnosis (years)
1	63	Dysesthesias and paresthesias in the hands and feet	38	Lymphomonocytic vasculitis	—	8
2	65	Paresthesias in hands and feet	18	Necrotizing Lymphomonocytic vasculitis	—	3.5
3	68	Dysesthesias and paresthesias in the hands and feet	23	Lymphomonocytic vasculitis	—	4
4	64	Paresthesias in the hands and feet	27	Necrotizing Lymphomonocytic vasculitis	—	2.6
5	73	Paresthesias and left foot drop	18	Necrotizing Lymphomonocytic vasculitis	ANA 1 : 160; no evidence of SLE or SS	3
6	56	Dysesthesias and paresthesias in both lower extremities	6	Necrotizing Lymphomonocytic vasculitis	ESR 40 mm/hrANA 1 : 80No evidence of SLE, SS, RA	3

ESR: erythrocyte sedimentation rate; ANA: antinuclear antibody; SLE: systemic lupus erythematosus; SS: Sjögren syndrome; RA: rheumatoid arthritis.
